# Surface Plasmon Resonance Sensor Based on Ethylene Tetra-Fluoro-Ethylene Hollow Fiber

**DOI:** 10.3390/s151127917

**Published:** 2015-11-03

**Authors:** Pan Chen, Yu-Jing He, Xiao-Song Zhu, Yi-Wei Shi

**Affiliations:** 1 School of Information Science and Engineering, Fudan University, 220 Handan Road, Shanghai 200433, China; E-Mails: kaxmail@yeah.net (P.C.); Arcadia_H@yeah.net (Y.-J.H.); ywshi@fudan.edu.cn (Y.-W.S.); 2 Key Laboratory for Information Science of Electromagnetic Wave (MoE), Fudan University, 220 Handan Road, Shanghai 200433, China

**Keywords:** surface plasmon resonance, hollow fiber, sensor, ethylene tetra-fluoro-ethylene

## Abstract

A new kind of hollow fiber surface plasmon resonance sensor (HF-SPRS) based on the silver-coated ethylene tetra-fluoro-ethylene (ETFE) hollow fiber (HF) is presented. The ETFE HF-SPRS is fabricated, and its performance is investigated experimentally by measuring the transmission spectra of the sensor when filled by liquid sensed media with different refractive indices (RIs). Theoretical analysis based on the ray transmission model is also taken to evaluate the sensor. Because the RI of ETFE is much lower than that of fused silica (FSG), the ETFE HF-SPRS can extend the lower limit of the detection range of the early reported FSG HF-SPRS from 1.5 to 1.42 approximately. This could greatly enhance the application potential of HF-SPRS. Moreover, the joint use of both ETFE and FSG HF-SPRSs can cover a wide detection range from 1.42 to 1.69 approximately with high sensitivities larger than 1000 nm/RIU.

## 1. Introduction

The surface plasmon resonance (SPR) technique has been extensively studied for its high refractive index (RI) sensitivity since the 1960s, when it was first released. Many kinds of sensors based on the SPR technique have been developed and used in the area of chemical and biomedical sensing in recent years. Bimolecular interactions and chemical reactions can be specified by monitoring the accompanying RI changes [1,2,3,4]. Most of these SPR sensors (SPRSs) are prism based and fiber based. In particular, fiber SPRSs have attracted much interest in recent years for their advantages, such as easy coupling, small volume and remote sensing [5,6,7].

As a kind of optical waveguide, hollow fiber (HF) has been widely studied in recent years for its high power threshold and low transmission losses in the infrared and visible region. Furthermore, taking advantage of the hollow core in its structure, HF can act as an optical waveguide and a sensing cell simultaneously. It found applications in the field of sensing, such as infrared absorption and Raman scattering [8,9,10,11,12,13]. In 2013, the HF-SPRS, which was fabricated by depositing a silver film with a thickness less than 100 nm on the inner wall of a fused silica (FSG) capillary tube, was reported [14]. This sensor has comparable sensitivity to other fiber SPRS, while its figure of merit (FOM) is not so high. Later, a kind of HF long-range SPR (LRSPR) sensor based on dielectric/silver-coated HF was reported [15]. Comparing to the HF-SPRS, the HF LRSPR sensor was significantly enhanced five times approximately in the FOM without decreasing in sensitivity. However, both of these sensors are based on fused silica HF, so they cannot detect a medium with an RI lower than fused silica. In other words, these HF-SPRSs are suitable for the detection of medium with a high RI because of their high RI detection range, which is above 1.5 approximately. Since aqueous solutions with lower RIs are more common in biomedical sensing, the HF-SPRS will be more useful if its RI detection range can be shifted towards a lower RI direction to match the aqueous solutions.

Changing the tube material of the HF from fused silica to some other materials with lower RIs is a feasible solution to extend the detection range of the HF-SPRS to a lower RI range. In this paper, we present a new kind of ethylene tetra-fluoro-ethylene (ETFE) HF-SPRS, which adopts ETFE instead of FSG as the base tube material. The ETFE tube is more flexible and less fragile than the FSG tube, which makes it safer in medical applications and more conducive to miniaturization of the instruments. More importantly, polymers with low RIs, such as Teflon AF, PTFE and other series, are very difficult to coat with a metal film because of their non-stick property, while ETFE has a relatively strong ability of metal adhesion. An ETFE HF-SPRS with a lower RI detection range than the FSG HF-SPRS was fabricated. Its performance was investigated experimentally by measuring the transmission spectra of the sensor. Theoretically analysis based on the ray transmission model was also carried out. The presented sensor shows the capability to extend the detection range to a lower RI range, which could greatly enhance the application potential of the HF-SPRS.

## 2. Theory

### 2.1. Theoretical Model

**Figure 1:**
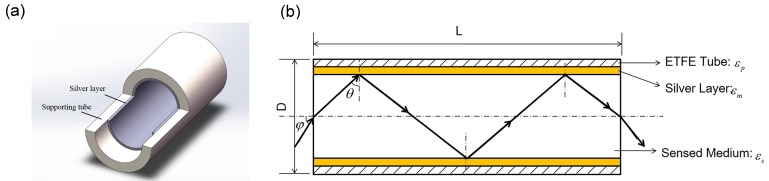
Schematic diagram of ethylene the tetra-fluoro-ethylene (ETFE) hollow fiber surface plasmon resonance sensor (HF-SPRS): (**a**) structure of the sensor; (**b**) lengthwise section of the ray transmission model.

The structure of the ETFE HF-SPRS is shown in Figure 1a. A thin silver film is coated on the inner surface of the ETFE tube. The sensed liquid medium with an RI higher than ETFE is filled in the hollow core of the HF. If the thickness of the silver layer is small enough (about less than 100 nm), surface plasmon waves would be excited on the silver-ETFE interface when appropriate light transmits in the ETFE HF-SPRS. SPR would demonstrate itself as a sharp dip in the transmission spectra of the sensor. The wavelength where the SPR dip is located is called the resonance wavelength (RW). Therefore, the slight change of the RI of the sensed liquid medium filled in the HF, which may represent some important physical and chemical properties, can be detected by measuring the shift of the RW in the transmission spectra.

As shown in Figure 1b, a ray transmission model is established to analyze the properties of the HF-SPRS theoretically [16,17]. The transmission of an incident meridian ray in the HF sensor is shown in the figure. Besides the transmission loss of the incident light, the total loss in the HF-SPRS also includes material loss of the analyte, input loss at the inlet and output loss at the outlet. However, these three losses in our experiments are almost equivalent for all wavelengths in the visible regions because the liquid sensed media are transparent. Therefore, these three losses show up as direct current components in the transmission spectra. The SPR effect demonstrates itself as a sharp dip in the transmission spectrum; while the RW of the SPR dip, which we are really interested in and which relates to the sensor’s performance, could not be influenced by the absolute values of these three losses. Here, we use the transmission loss of meridian rays to approximate the total loss when the incident angle is small enough. The reason for this approximation has been discussed in [17].

Only p-polarized light (*i.e.*, transverse magnetic (TM) mode) is taken into consideration in the calculation, because s-polarized light cannot excite SPR here. The p-polarized reflection on the inner surface of HF can be calculated with the three-layer (liquid sensed medium/silver/ETFE) Fresnel equation [18].

(1) $$R_{p}\left(\theta \right)={\left|\frac{r_{sm}+r_{mp}\exp\left(2ik_{mz}d\right)}{1+r_{sm}r_{mp}\exp\left(2ik_{mz}d\right)}\right|}^{2}$$

(2) $$r_{sm}=\frac{k_{sz}\varepsilon_{m}-k_{mz}\varepsilon_{s}}{k_{sz}\varepsilon_{m}+k_{mz}\varepsilon_{s}}$$

(3) $$r_{mp}=\frac{k_{mz}\varepsilon_{p}-k_{pz}\varepsilon_{m}}{k_{mz}\varepsilon_{p}+k_{pz}\varepsilon_{m}}$$

(4) $$k_{jz}={\left(\varepsilon_{j}\frac{\omega^{2}}{c^{2}}-k_{x}^{2}\right)}^{1/2},j=s,m,p$$

(5) $$k_{x}=\sqrt{\varepsilon_{s}}\frac{\omega }{c}\sin\theta$$

where *k_sz_*, *k_mz_*, *k_pz_* are the propagation constants in the liquid sensed medium, silver layer and base tube, respectively. *k_x_* is the propagation constant of the incident wave. Furthermore, *ε_s_*, *ε_m_*, *ε_p_* are the dielectric constants of the sensed medium, silver film and ETFE tube, respectively. *d* is the silver layer thickness; *ω* is the frequency of the incident light; and *c* is the speed of light in a vacuum.

The distribution of the input power of incident light coupled to the sensor is close to Gaussian with profile *P*_0_(*φ*), which can be expressed approximately as [17]:

(6) $$P_{0}\left(\varphi \right)\propto \exp\left(-\frac{\varphi^{2}}{\varphi_{0}^{2}}\right)$$

where *φ* is the launching angle of the input light and *φ*_0_ depends on the divergence of the input light. In our experiment setup, the angle of the input light *φ*_0_ is related to the wavelength of the incident light *λ*. The dispersion of *φ*_0_ is measured as *φ*_0_(*λ*) = −0.01*λ* + 12.76 [14]. The relationship between angle *φ* and *θ*, shown in Figure 1, follows Snell’s law:

(7) $$\sin\varphi =n_{s}\cos\theta$$

where *n_s_* is the RI of the sensed medium. Hence, the power of the output light is given as:

(8) $$P=\int_{\theta_{cr}}^{\pi /2}P_{0}\left(\theta \right)R_{p}{\left(\theta \right)}^{N(\theta )}d\theta$$

where *P*_0_(*θ*) is the input power of incident light, *N*(*θ*) is the reflection times of the light and *θ_cr_* is the critical angle of total reflection on the interface, which are:

(9) $$P_{0}\left(\theta \right)\propto \exp\left(-\frac{\arcsin^{2}\left(n_{s}\cos\theta \right)}{\varphi_{0}^{2}}\right)$$

(10) $$N\left(\theta \right)=\frac{L}{D\tan\theta }$$

(11) $$\theta_{cr}=arcsin\left(n_{p}/n_{s}\right)$$

*L* and *D* are the length and diameter of the sensor, respectively; *n_p_* is the RI of the ETFE tube. Therefore, the generalized expression for the normalized transmittance of the HF-SPRS is:

(12) $$T=\frac{\int_{\theta_{cr}}^{\pi /2}P_{0}\left(\theta \right)R_{p}{\left(\theta \right)}^{N(\theta )}d\theta }{\int_{\theta_{cr}}^{\pi /2}P_{0}\left(\theta \right)d\theta }$$

In this paper, the dielectric constant of the silver layer in the sensor is obtained by the Drude free electron model [19]:

(13) $$\varepsilon \left(\lambda \right)=\varepsilon_{r}+i\varepsilon_{i}=1-\frac{\lambda^{2}\lambda_{c}}{\lambda_{p}^{2}\left(\lambda_{c}+i\lambda \right)}$$

where, *λ_p_* = 1.4541 × 10^−7^ m, *λ_c_* = 1.7614 × 10^−5^ m.

### 2.2. Sensor Performance

Sensitivity is an important parameter to evaluate the performance of an SPRS. Usually, the sensitivity of a wavelength-interrogated SPRS is defined as:

(14) $$S_{n}=\Delta \lambda_{res}/\Delta n_{s}$$

where Δ*λ_res_* is the shift of RW when the RI of the sensed medium changes Δ*n_s_*. In addition, the full width at half maximum (FWHM) of the resonance dip in the SPR spectrum, which can influence the measuring accuracy of the RW, is also an important factor for the sensor’s performance. Smaller FWHM leads to higher detection accuracy of the sensor. Furthermore, the figure of merit (FOM), a more comprehensive parameter to evaluate the SPRS’s performance, is defined as [20]:

(15) $$FOM=S_{n}/FWHM$$

## 3. Fabrication and Experimental Setup

**Figure 2:**
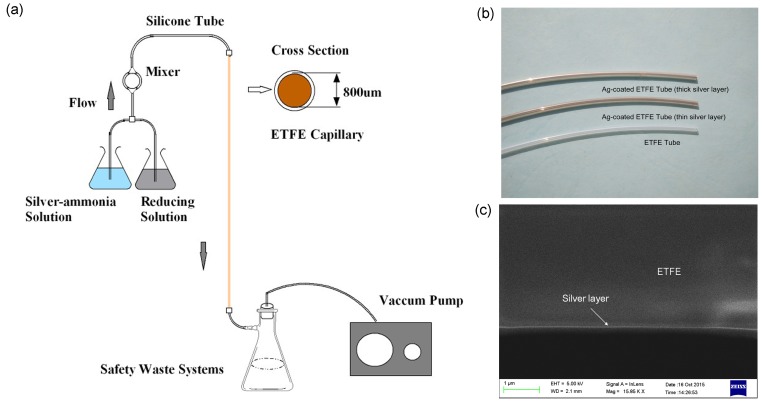
(**a**) Schematic diagram of the liquid phase coating method for silver plating; (**b**) ETFE tube and two Ag-coated ETFE tubes with different Ag film thicknesses; (**c**) SEM picture of the cross-section of the HF used in the experiment.

The essential procedure to fabricate ETFE HF-SPRS is coating a smooth and uniform silver film with a thickness less than 100 nm on the inner surface of the ETFE tube. Comparing to FSG, ETFE has a much lower RI, which can shift the sensor’s detection range to a lower RI range. However, coating of a silver film in the ETFE tube is not as easy as doing that in the FSG tube, although ETFE has much stronger metal adhesion than other fluoropolymers. There are several kinds of approaches, such as electrochemical deposition [21] and electrophoretic deposition [22], that could give a homogenous silver coating. In our experiments, we use an improved liquid phase deposition method, as shown in Figure 2a, to deposit the silver film on the inner surface of the ETFE tube. Pumped by a vacuum pump, the prepared silver-ammonia and glucose solutions are fully mixed in the mixer and then pass through the ETFE tube. Silver particles will deposit on the inner surface of the tube and form a silver film gradually. The deposition temperature, time and speed of solution passing through the tube are the key factors affecting the quality of the silver film. A high temperature causes difficulties in controlling the silver layer thickness, while a low temperature leads to bad silver layer quality. The deposition time influences the silver layer thickness, and the solution speed affects the film quality. Experiments show that the coating procedure in the ETFE tube should have a little bit lower temperature and faster solution speed than that in the FSG tube to achieve better film quality. Moreover, because the growth rate of the silver film is slower in the ETFE tube than in FSG tube, it costs more time to get the same silver film thickness. A 20 cm-long ETFE HF with an 800-μm diameter was fabricated at a temperature of 12 °C. The deposition time of the silver layer was 90 s, and the solution speed is 28 mL/min. A piece of fiber with a short length of about 5 cm is cut off as the SPRS used in the following spectra experiments.

In addition, the photo of the ETFE tube and two Ag-coated ETFE tubes with different Ag film thicknesses is shown as Figure 2b. This could convince one that Ag has been coated properly. The SEM photo of the Ag/ETFE interface of the cross-section of the Ag-coated ETFE tube was also taken and shown as Figure 2c. We have tried to obtain a photo with larger magnification, but this failed. Because of the charging effect and the low electrical conductivity of the ETFE tube, the image becomes even worse and too blurred to be distinguished if increasing the magnification further. The silver film can be seen clearly in the SEM photo, although its thickness cannot be measured because of the low magnification.

**Figure 3:**
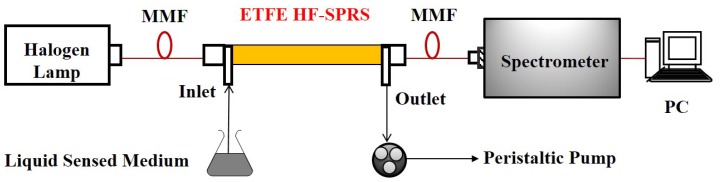
Diagram of the experimental setup.

Figure 3 shows the experimental system for measuring the SPR spectra. The light emitted from a halogen lamp was launched into the ETFE HF-SPRS via the multi-mode fiber (MMF) and then collected by the spectrometer (Horiba JobinYvon iHR550, Paris, France). In addition, to realize real-time detection, the liquid sensed medium was injected into the sensor by a peristaltic pump through an L-type joint connected with silicone tubes.

Two kinds of solutions were used as the sensed media to cover the RI range from 1.42 to 1.58. They are mixed solutions of deionized water and glycerin with different volume ratios for the RI range of 1.42 to 1.46, while mixed solutions of kerosene and polymethyl phenyl silicone fluid for an RI range above 1.46. The exact RIs of the solutions used in experiments were measured by the Abbey refractometer as 1.4287, 1.4328, 1.4413, 1.4506, 1.4605, 1.4701, 1.4808, 1.4904, 1.5003, 1.5102, 1.5190, 1.5367 and 1.5412, respectively.

## 4. Results and Discussion

**Figure 4:**
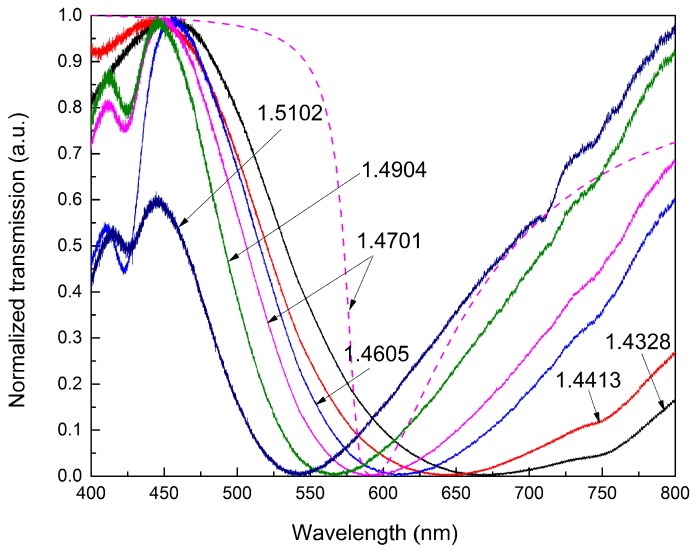
Normalized transmission spectra of ETFE HF-SPRS with different *n_s_*; the corresponding *n_s_* is labeled in the figure.

Figure 4a shows the normalized transmission spectra of the ETFE HF-SPRS for the liquid sensed media with different RIs. The RIs of the media are 1.4328, 1.4413, 1.4605, 1.4701, 1.4904 and 1.5102, respectively. The solid lines represent the measured spectra. As can be seen from the figure, the resonance dip in the spectrum shifts towards shorter wavelength when the RI of the sensed medium increases. In other words, the RW blue shifts with increased RI. The theoretical calculated spectrum with Equation (12) for *n_s_* = 1.4701 is also shown in the figure as the dashed line for comparison. It can be seen that the RW of the theoretical spectrum is close to the measured result, while the resonance dip is much narrower. The wider SPR dip of the measured spectra means that the experimental data have much larger FWHM than the theoretical results. This is mainly due to the non-uniformity of the silver layer. The SPR spectrum of a non-uniform silver layer can be simply seen as the combination of the SPR spectra of a set of silver layers with different thicknesses. Additionally, the RW of the SPR spectra will shift as the silver layer thickness varies. Therefore, the combination will definitely broaden the SPR dip.

**Figure 5:**
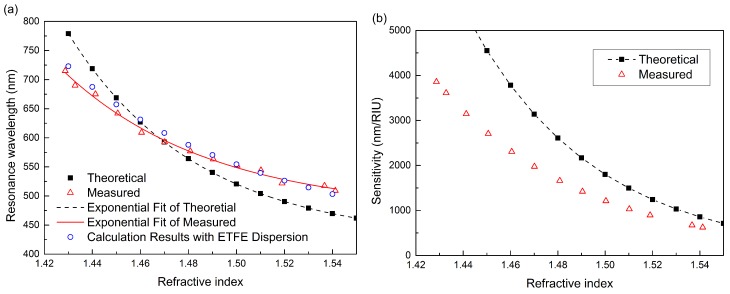
Theoretical and measured results of resonance wavelength (RW) and sensitivity for ETFE HF-SPRS. (**a**) RW *versus n_s_*; (**b**) sensitivity *versus n_s_*.

The RW of each experimental SPR spectrum for different sensed RI was measured, shown with red triangles in Figure 5a. Theoretical data of RWs obtained from the spectra calculated with Equation (12) are also shown in the figure with black squares for comparison, while the thickness of silver layer is 42 nm and the RI of ETFE tube in the calculation is about 1.3850 at a wavelength of 589 nm (measured by an Abbe refractometer).

The black dashed line represents exponential fitting results of the theoretical data. The fit function is *λ_res_* = 423.43324 + 1.18189 × 10^14^ exp(−18.55224*n_s_*), with a correlation coefficient of 0.99856. It can be seen that the exponential fitting matches very well with the data points. Therefore, we also use the exponential function to fit the experimental measured data. The red solid line is the result of exponential fitting. The fit function is *λ_res_* = 407.6446 + 2.64321 × 10^12^ exp(−16.18922*n_s_*), and the correlation coefficient is 0.99379. The lines of theoretical simulations and measured results have approximately the same trend. The deviation is mainly because of the dispersion of ETFE and sensed media, which is not taken into consideration in the calculation with Equation (12). Here, we assume that the RI of ETFE is expressed according to the Cauchy equation as:

(16) $$n_{p}^{2}=1.344+0.01785/\lambda^{2}-0.00065/\lambda^{4}$$

This dispersion is similar to the dispersion curve in [23]. Although it may have a small difference in values, we could use it to qualitatively illustrate the influence of the dispersion of ETFE on the theoretical results. The calculation results of taking the above dispersion into consideration are shown in Figure 5a as the blue circles. It can be seen that the absolute slope rate of the theoretical curve decreases and is very close to the experimental curve. This means that the deviation of RW between experimental and theoretical results is mainly due to the material dispersion. Because the assumed dispersion of ETFE is not strictly accurate, the later theoretical analyses still use the non-dispersion results. Theoretically, the sensor’s detection range could cover any RI higher than the the ETFE tube. However, it is also limited because of the finite wavelength range of the light source and spectrometer. For example, if the RI of the sensed medium is lower than 1.42, the resonance dip will go beyond 800 nm, which is the upper limit of the spectrometer in our experiments. Thus, the efficient detection range of the ETFE HF-SPRS in our experiments is approximately from 1.42 to 1.54.

According to Equation (14), the definition of the sensitivity, the first derivatives of the curves of RW *versus n_s_* in Figure 5a are the sensitivities of the sensor. The absolute values of the calculated sensitivities for both theoretical and experimental data are shown in Figure 5b. It can be seen that the sensor has a higher sensitivity in the lower RI region, while the RW is located in the longer wavelength region. Additionally, the sensitivity decreases gradually from about 4000 nm/RIU to 600 nm/RIU as the RI of sensed medium increases from 1.42 to 1.54.

**Figure 6:**
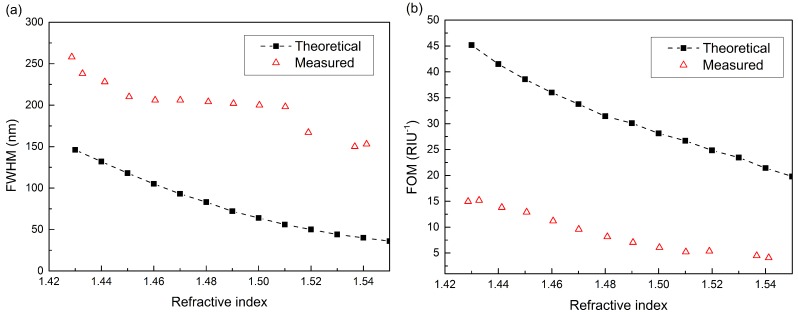
Comparison of measured transmission spectra and theoretical results with different *n_s_*. (**a**) FWHM *versus n_s_*; (**b**) figure of merit (FOM) *versus n_s_*.

Besides sensitivity, the detection accuracy is another important parameter for evaluating the performance of the SPRS [24]. Additionally, the FWHM of the resonance dip is the major factor that affects the detection accuracy. A large FWHM would increase the difficulty to determine the RW accurately and leads to a larger deviation. Figure 6a shows the FWHMs of the resonance dips for the ETFE HF-SPRS, both theoretically and experimentally. Generally, the experimentally-measured FWHMs are larger than the theoretical data, which could be clearly seen in Figure 4. The result is consistent with the conclusions in [14,15,25]. Coating with a more uniform silver layer by improving the coating method can decrease the FWHM effectively. Moreover, adopting a light source with a smaller divergence angle will also contribute to a smaller FWHM [14].

With the FWHM data in Figure 6a, the FOM of the sensor was calculated with Equation (15) and is shown in Figure 6b. The experimental FOM decreases gradually from more than 15 to 4 as the sensed RI increases from 1.42 to 1.54. Additionally, it is smaller than the theoretical value because of the larger FWHM.

**Figure 7:**
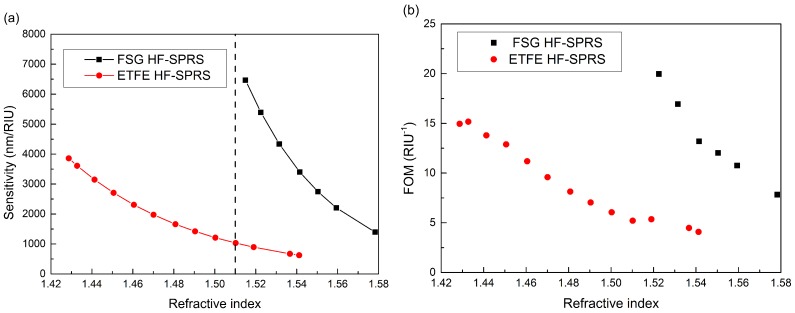
(**a**) Sensitivities of ETFE and FSG HF-SPRS: using the ETFE HF-SPRS and FSG HF-SPRS in the refractive index (RIs) range below and above 1.51, respectively; (**b**) FOMs *versus n_s_*.

The main contribution of the ETFE HF-SPRS is to extend the lower limit of the RI detection range of HF-SPRS approximately from 1.51 to 1.42. Although the sensor’s detection range can be up to 1.54, its performance decreases quickly as the sensed RI increases. Utilizing the ETFE HF-SPRS in the RI range above 1.51 is not a good choice. Thus, the joint use of both ETFE HF-SPRS and FSG HF-SPRS to get high performance in the whole detection range is optimal. Figure 7a,b shows the sensitivities and FOMs of both ETFE HF-SPRS and FSG HF-SPRS in the wide RI range from 1.42 to 1.58. The data of the FSG HF-SPRS are from [14], when the silver layer thickness is 57 nm. If 1.51 is chosen to be the boundary of the detection range of the two sensors, *i.e.*, using the ETFE HF-SPRS and the FSG HF-SPRS in the RI range below and above 1.51, respectively, we can achieve optimal sensitivities larger than 1000 nm/RIU and FOMs larger than 5 RIU^−1^ in the whole RI range above 1.42. The parameter details of both sensors and their joint use are also listed in Table 1. The sensitivity and FOM ranges listed in the table are the maximum and minimum values of experimental data, but not the exact data corresponding to the upper and lower limits of the RI ranges. Therefore, the joint use of ETFE and FSG HF-SPRSs has a wide detection range and a high sensitivity comparable to the theoretical values reported for cladding-off cylindrical fiber (3000 nm/RIU) [26], tapered fiber (2700–4900 nm/RIU) [27], long period grating fiber (817 nm/RIU) [28] and selected coated photonic crystal fiber (PCF) (maximum of 5500 nm/RIU) [29]. The hollow core fiber sensor assisted by a fiber Bragg grating can also detect the RI of the sensed medium filled in the hollow core [30]. This sensor has a lower sensitivity 230 nm/RIU, but a fairly high resolution because of the narrow reflection spectrum of the fiber Bragg grating. However, the sensor is a theoretical model and may encounter some difficulties in fabrication and the injection of the sensed medium, because of its small bore size, which is only several microns.

**Table 1:** Characteristics of the HF-SPRS.

Structure	Wavelength (nm)	RI Detection Range	Sensitivity (nm/RIU)	FOM (RIU^−1^)
ETFE HF-SPRS	400 to 800	1.42 to 1.54	624 to 3858	4 to 15
FSG HF-SPRS	400 to 800	1.51 to 1.58	1189 to 6607	12 to 25
ETFE and FSG	400 to 800	1.42 to 1.58	1031 to 6607	5 to 25

## 5. Conclusions

We have proposed a new kind of SPRS based on ETFE HF, which could greatly extend the detection range of HF-SPRS towards the lower RI direction. Compared to the FSG HF-SPRS, the ETFE HF-SPRS is more flexible and has a similar sensitivity, but moves the lower limit of RI detection range from 1.5 to 1.42 approximately. The performance of the fabricated ETFE HF-SPRS was investigated both theoretically and experimentally. The joint use of ETFE and FSG HF-SPRSs has a wide detection range (1.42 to 1.69) with high sensitivities larger than 1000 nm/RIU in the whole range. With the extension in the detection range, the application potentiality of the HF-SPRS could be greatly enhanced. Additionally, it would be further improved if other kinds of materials with even lower RI than ETFE could be used as the supporting tube material to fabricate the HF-SPRS.

## References

[1] Nylander C., Liedberg B., Lind T. (1983). Gas detection by means of surface plasmon resonance. Sens. Actuators.

[2] Liedberg B., Nylander C., Lunstrom I. (1983). Surface plasmon resonance for gas detection and biosensing. Sens. Actuators.

[3] Jorgenson R., Yee S. (1993). A fiber-optic chemical sensor based on surface plasmon resonance. Sens. Actuators B Chem..

[4] Liedberg B., Nylander C., Lundstrom I. (1995). Biosensing with surface plasmon resonance how it all started. Biosens. Bioelectron..

[5] Fallauto C., Liu Y., Perrone G., Vallan A. (2014). Compensated surface plasmon resonance sensor for long-term monitoring applications. Instrumentation and Measurement. IEEE Trans. Instrum. Meas..

[6] Chen X., Luo Y., Xu M., Zhang Y., He Y., Tang J., Yu J., Zhang J., Chen Z. (2014). Refractive index and temperature sensing based on surface plasmon resonance fabricated on a side-polished fiber. Acta Opt. Sin..

[7] Yan P.G., Xing F.F. (2009). Microstructured-optical fiber surface-plasm on-resonance sensor. J. Shenzhen Univ. Sci. Eng..

[8] Hidaka T., Morikawa T., Shimada J. (1981). Hollow-core oxide-glass cladding optical fibers for middle-infrared region. J. Appl. Phys..

[9] Cadarso V., Llobera A., Fernandez-Sanchez C., Darder M., Dominguez C. (2009). Hollow waveguide-based full-field absorbance biosensor. Sens. Actuators B Chem..

[10] Kim S.S., Menegazzo N., Young C., Chan J., Carter C., Mizaikoff B. (2009). Mid-infrared trace gas analysis with single-pass Fourier transform infrared hollow waveguide gas sensors. Appl. Spectrosc..

[11] Zhao H., Jiang Y., Luo T. (2012). Hollow optical fiber sensor based on surface plasmon resonance. Acta Opt. Sin..

[12] Sun Y.J., Zeng X., Liu B.H., Shi W.W. (2013). Fabrication of Low-Loss AgI/Ag Hollow Fibers for Laser Light Delivery in the Near Infrared Region. Acta Opt. Sin..

[13] Sheng X., Chen G., Shi Y. (2014). Novel Chemiluminometric Sensing System Based on Hollow Fiber. Acta Opt. Sin..

[14] Liu B.H., Jiang Y.X., Zhu X.S., Tang X.L., Shi Y.W. (2013). Hollow fiber surface plasmon resonance sensor for the detection of liquid with high refractive index. Opt. Express.

[15] Jiang Y.X., Liu B.H., Zhu X.S., Tang X.L., Shi Y.W. (2015). Long-range surface plasmon resonance sensor based on dielectric/silver coated hollow fiber with enhanced figure of merit. Opt. Lett..

[16] Sharma A.K., Gupta B. (2006). Theoretical model of a fiber optic remote sensor based on surface plasmon resonance for temperature detection. Opt. Fiber Technol..

[17] Matsuura Y., Hongo A., Saito M., Miyagi M. (1989). Loss characteristics of circular hollow waveguides for incoherent infrared light. JOSA A.

[18] Born M., Wolf E. (1999). Principles of Optics: Electromagnetic Theory of Propagation, Interference and Diffraction of Light.

[19] Drude P. (1900). Zur elektronentheorie der metalle; II. Teil. galvanomagnetische und thermomagnetische effecte. Ann. Phys..

[20] Shalabney A., Abdulhalim I. (2012). Figure-of-merit enhancement of surface plasmon resonance sensors in the spectral interrogation. Opt. Lett..

[21] Yang S., Lapsley M.I., Cao B., Zhao C., Zhao Y., Hao Q., Kiraly B., Scott J., Li W., Wang L. (2013). Large-Scale Fabrication of Three-Dimensional Surface Patterns Using Template-Defined Electrochemical Deposition. Adv. Funct. Mater..

[22] Yang S., Cai W., Kong L., Lei Y. (2010). Surface Nanometer-Scale Patterning in Realizing Large-Scale Ordered Arrays of Metallic Nanoshells withell-Defined Structures and Controllable Properties. Adv. Funct. Mater..

[23] French R.H., Rodríguez-Parada J.M., Yang M.K., Derryberrya R.A., Lemon M.F., Brown M.J., Haeger C.R., Samuels S.L., Romano E.C., Richardson R.E. (2011). Optical properties of polymeric materials for concentrator photovoltaic systems. Sol. Energy Mater. Sol. Cells.

[24] Shalabney A., Abdulhalim I. (2011). Sensitivity-enhancement methods for surface plasmon sensors. Laser Photonics Rev..

[25] Suzuki H., Sugimoto M., Matsui Y., Kondoh J. (2008). Effects of gold film thickness on spectrum profile and sensitivity of a multimode-optical-fiber SPR sensor. Sens. Actuators B Chem..

[26] Sharma A.K., Gupta B. (2006). Fibre-optic sensor based on surface plasmon resonance with Ag-Au alloy nanoparticle films. Nanotechnology.

[27] Verma R.K., Sharma A.K., Gupta B. (2008). Surface plasmon resonance based tapered fiber optic sensor with different taper profiles. Opt. Commun..

[28] Yu X., Zhang Y., Pan S., Shum P., Yan M., Leviatan Y., Li C. (2010). A selectively coated photonic crystal fiber based surface plasmon resonance sensor. J. Opt..

[29] Xia L., Zhang Y., Zhou C., Liu D. (2011). Numerical analysis of plasmon polarition refractive index fiber sensors with hollow core and a long period grating. Opt. Commun..

[30] Nemova G., Kashyap R. (2006). Modeling of Plasmon-Polariton Refractive-Index Hollow Core Fiber Sensors Assisted by a Fiber Bragg Grating. J. Light. Technol..

